# Association of Triglyceride-to-HDL-C Ratio, Triglyceride–Glucose Index, and Inflammatory Biomarkers with Mortality in Intensive Care Unit Patients with Sepsis

**DOI:** 10.3390/diagnostics16060844

**Published:** 2026-03-12

**Authors:** Nilgün Şahin, Semih Aydemir, Nazan Has Selmi, İbrahim Ertaş, Yavuz Kutay Gökçe, Cihan Döğer, Gökçen Terzi, Mesher Ensarioğlu

**Affiliations:** 1 Department of Anesthesiology and Reanimation, Ankara Guven Hospital, 06540 Ankara, Türkiye; 2 Department of Critical Care, Yıldırım Beyazıt University, Yenimahalle Training and Research Hospital, 06010 Ankara, Türkiye; drsemihaydemir@gmail.com (S.A.); nazanhasselmi3@gmail.com (N.H.S.); 3 Department of Emergency Medicine, Yıldırım Beyazıt University, Yenimahalle Training and Research Hospital, 06010 Ankara, Türkiye; ertasibrahim0880@gmail.com; 4 Department of Internal Medicine, Yıldırım Beyazıt University, Yenimahalle Training and Research Hospital, 06010 Ankara, Türkiye; ykgokce06@gmail.com; 5 Department of Anesthesiology and Reanimation, Ankara City Hospital, 06800 Ankara, Türkiye; cihandoger@gmail.com; 6 Department of Anesthesiology and Reanimation, Yıldırım Beyazıt University, Yenimahalle Training and Research Hospital, 06010 Ankara, Türkiye; gokcenatilla2@gmail.com; 7 Department of Anesthesiology and Reanimation, Health Sciences University, Gülhane Training and Research Hospital, 06010 Ankara, Türkiye; meshercapras@gmail.com

**Keywords:** sepsis, triglyceride–glucose index, TG/HDL-C ratio, pan-immune-inflammation value, mortality, intensive care unit, prognosis

## Abstract

**Background/Objectives**: This study aimed to investigate the prognostic significance of the triglyceride–glucose index (TGI), triglyceride-to-high-density lipoprotein cholesterol (TG/HDL-C) ratio, and inflammatory biomarkers in predicting short-term mortality among intensive care unit (ICU) patients with sepsis. Additionally, this study evaluated whether combining these indices with conventional clinical scores improves prognostic accuracy. **Methods**: This retrospective cohort study included 600 adult ICU patients diagnosed with sepsis according to Sepsis-3 criteria between January 2020 and April 2025. Clinical, biochemical, and hematological data were collected within the first 24 h of ICU admission. Metabolic indices (TGI, TG/HDL-C) and inflammatory markers (neutrophil-to-lymphocyte ratio [NLR], systemic immune-inflammation index [SII], and pan-immune-inflammation value [PIV]) were analyzed. The primary outcome was 28-day mortality. Receiver operating characteristic (ROC) analyses, Kaplan–Meier survival curves, and a multivariable logistic regression model were applied to determine prognostic performance. **Results**: Non-survivors exhibited significantly higher levels of TGI, TG/HDL-C, NLR, SII, and PIV compared to survivors (all *p* < 0.001). In ROC analysis, TGI (AUC = 0.75, 95% CI: 0.71–0.79), TG/HDL-C (AUC = 0.72, 95% CI: 0.68–0.76), and PIV (AUC = 0.78, 95% CI: 0.74–0.82) demonstrated good discriminative power for predicting 28-day mortality. Multivariate logistic regression identified TGI > 8.95 (OR = 1.44, 95% CI: 1.19–1.74, *p* < 0.001), TG/HDL-C > 3.95 (OR = 1.31, 95% CI: 1.08–1.59, *p* = 0.005), and PIV > 260 (OR = 1.49, 95% CI: 1.22–1.82, *p* < 0.001) as independent predictors of mortality. Integrating TGI and PIV with the SOFA score improved prognostic performance (ΔAUC = +0.04). **Conclusions**: Both TGI and TG/HDL-C are independent predictors of short-term mortality in septic ICU patients, reflecting the contribution of metabolic dysregulation to disease severity. The PIV demonstrated comparable predictive ability to conventional severity scores. Combining metabolic and inflammatory biomarkers with established clinical indices may enhance early risk stratification and guide personalized management strategies in sepsis.

## 1. Introduction

Sepsis remains one of the leading causes of morbidity and mortality in intensive care units (ICUs) worldwide, representing a major global health challenge despite advances in antimicrobial therapy and organ support modalities. It is characterized by life-threatening organ dysfunction resulting from a dysregulated host response to infection, with mortality rates ranging from 25% to over 50% depending on disease severity and comorbid conditions [1,2]. Early identification of high-risk patients and rapid risk stratification are essential for guiding timely interventions and optimizing resource allocation in critically ill populations [3].

Metabolic disturbances are common in sepsis, reflecting the combined effects of systemic inflammation, endocrine dysregulation, and critical illness–related catabolism. Dyslipidemia and stress-induced hyperglycemia are particularly prominent metabolic features, both of which have been linked to immune dysfunction, endothelial injury, and adverse outcomes [4,5]. The triglyceride–glucose index (TGI) and triglyceride-to-high-density lipoprotein cholesterol (TG/HDL-C) ratio are simple, cost-effective, and readily available surrogate markers of insulin resistance and atherogenic dyslipidemia [6,7,8,9,10,11,12,13,14,15,16,17]. These indices have shown prognostic value in various cardiometabolic and inflammatory conditions, including acute coronary syndromes, stroke, and critical illness [11,12,13,14,18,19].

In addition to metabolic indices, inflammatory biomarkers have been widely investigated as prognostic tools in sepsis. The neutrophil-to-lymphocyte ratio (NLR) and systemic immune-inflammation index (SII) are established predictors of outcomes in critically ill patients [20,21,22,23,24,25,26,27,28,29]. More recently, the Pan-Immune-Inflammation Value (PIV), calculated as (neutrophil × platelet × monocyte)/lymphocyte, has been proposed as a novel composite marker that integrates three major inflammatory cell lines [30,31,32,33,34]. PIV has demonstrated potential in predicting mortality across different clinical settings, but its role in sepsis remains underexplored [30,31,33].

Although several studies have investigated TGI, TG/HDL-C, and inflammatory markers in cardiovascular and metabolic disorders, their prognostic value in sepsis-specific populations remains poorly characterized. Furthermore, it is unclear whether metabolic indices perform as well as, or better than, inflammatory parameters such as NLR, SII, and PIV in predicting sepsis-related mortality.

We aimed to investigate the association between the TGI, TG/HDL-C ratio, and short-term mortality in ICU patients with sepsis. Specifically, we sought to determine whether these metabolic indices independently predict 28-day and in-hospital mortality; compare their prognostic performance with established inflammatory markers, including NLR, SII, and PIV; and evaluate whether incorporating these parameters into conventional clinical risk scores could enhance prognostic accuracy.

## 2. Materials and Methods

### 2.1. Study Design and Setting

This retrospective observational cohort study was conducted in the adult Intensive Care Unit (ICU) of Ankara City Hospital, a tertiary referral center with a capacity of 700 ICU beds, between 1 January 2020 and 30 April 2025. The ICU admits patients with various medical and surgical conditions, including those with sepsis and septic shock. The study protocol adhered to the Strengthening the Reporting of Observational Studies in Epidemiology (STROBE) guidelines.

This study was approved by the Ethics Committee of the Health Sciences University. The Declaration of Helsinki protocol was followed (approval: 2025/148; date: 23 September 2025). Due to the retrospective nature of this study, the requirement for informed consent was waived by the ethics committee.

Patients aged 18 years or older who were diagnosed with sepsis according to the Third International Consensus Definitions for Sepsis and Septic Shock (Sepsis-3) criteria—defined as a documented or suspected infection with an increase of ≥2 points in the Sequential Organ Failure Assessment (SOFA) score from baseline—were eligible for inclusion. Additional requirements were ICU admission within 24 h of meeting sepsis criteria and availability of fasting blood samples for triglyceride (TG), high-density lipoprotein cholesterol (HDL-C), and fasting plasma glucose (FPG) measurements obtained within the first 24 h of ICU admission. Patients were excluded if they were pregnant, had pre-existing advanced liver disease (Child–Pugh class C) or acute pancreatitis at admission, had lipid profile measurements obtained in a non-fasting state or beyond 24 h after ICU admission, were receiving high-dose corticosteroids or lipid-lowering therapy that could not be accounted for in statistical adjustments, or had incomplete clinical or laboratory data necessary for analysis.

### 2.2. Data Collection and Outcome Measures

Demographic, clinical, and laboratory data were obtained from the hospital’s electronic medical records and the ICU database. Recorded variables included demographic characteristics (age, sex, and body mass index [BMI]) and comorbidities such as diabetes mellitus (DM), hypertension, chronic kidney disease (CKD), chronic obstructive pulmonary disease (COPD), ischemic heart disease, chronic liver disease, and malignancy. Clinical data encompassed the primary site of infection, presence of septic shock, requirement for mechanical ventilation (MV), use of vasopressors, need for renal replacement therapy (RRT), and length of ICU stay. Disease severity was assessed using the Acute Physiology and Chronic Health Evaluation II (APACHE II) score and the Sequential Organ Failure Assessment (SOFA) score, both calculated within the first 24 h of ICU admission. Laboratory parameters collected within the first 24 h included fasting plasma glucose (mg/dL), triglycerides (mg/dL), high-density lipoprotein cholesterol (HDL-C; mg/dL), total cholesterol, low-density lipoprotein cholesterol (LDL-C), lactate, C-reactive protein (CRP), procalcitonin (PCT), albumin, creatinine, complete blood count with differential (neutrophil, lymphocyte, monocyte, and platelet counts), and liver function tests.

**Calculation of Derived Indices:**

➢ **Triglyceride-to-HDL-C ratio (TG/HDL-C):** TG (mg/dL)/HDL-C (mg/dL).
➢ **Triglyceride–glucose index (TGI):** ln [TG (mg/dL) × FPG (mg/dL)/2].
➢ **Neutrophil-to-lymphocyte ratio (NLR):** neutrophil count/lymphocyte count.
➢ **Platelet-to-lymphocyte ratio (PLR):** platelet count/lymphocyte count.
➢ **Monocyte-to-lymphocyte ratio (MLR):** monocyte count/lymphocyte count.
➢ **Systemic immune-inflammation index (SII):** (platelet count × neutrophil count)/lymphocyte count.
➢ **Platelet inflammatory value (PIV):** (neutrophil count × platelet count × monocyte count)/lymphocyte count.

**Figure 1:**
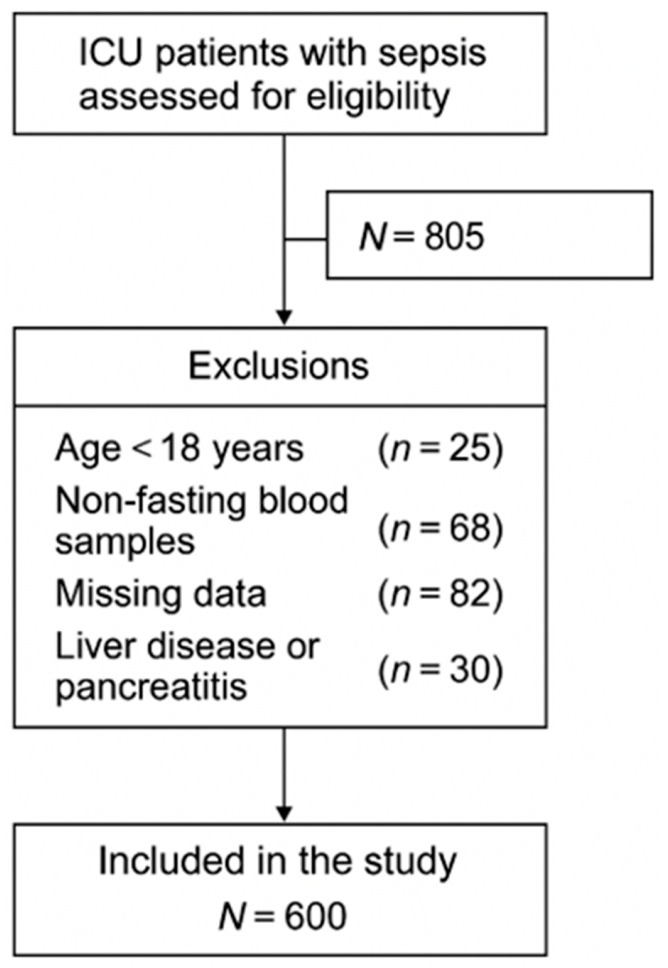
Flowchart of study.

The primary outcome of this study was 28-day all-cause mortality following ICU admission. Secondary outcomes included in-hospital all-cause mortality, length of ICU stay (days), duration of mechanical ventilation (days), and duration of vasopressor use (days) (Figure 1).

### 2.3. Statistical Analysis

Statistical analyses were performed using IBM SPSS Statistics for Windows, version 27.0 (IBM Corp., Armonk, NY, USA), and R software version 4.3. Continuous variables were tested for normality using the Shapiro–Wilk test. Normally distributed variables were expressed as the mean ± standard deviation (SD), while non-normally distributed variables were expressed as the median with interquartile range (IQR). Categorical variables were presented as counts and percentages.

Between-group comparisons were conducted using the student’s *t*-test or Mann–Whitney *U* test for continuous variables and the chi-square test or Fisher’s exact test for categorical variables, as appropriate. Receiver operating characteristic (ROC) curve analysis was used to evaluate the discriminative performance of metabolic and inflammatory biomarkers for predicting 28-day mortality. The area under the ROC curve (AUC) with 95% confidence intervals (CIs) was calculated, and optimal cut-off values were determined using the Youden index.

To identify independent predictors of 28-day mortality, univariate logistic regression analyses were first performed. Variables with *p* < 0.10 in univariate analyses were considered for inclusion in the multivariable logistic regression model. In addition, clinically relevant covariates known to influence sepsis outcomes (e.g., major comorbidities and disease severity indicators) were also evaluated. To prevent model overfitting and multicollinearity, the final multivariable model included only variables that were both clinically meaningful and statistically stable. Continuous biomarkers were retained in regression models to avoid information loss associated with dichotomization. Cut-off values derived from ROC analysis were used only for Kaplan–Meier survival visualization. Model discrimination was evaluated using ROC curve analysis and AUC estimates. Internal validation of model performance was performed using bootstrap resampling with 1000 iterations, and optimism-corrected AUC values were calculated. Model calibration was assessed using the Hosmer–Lemeshow goodness-of-fit test. Kaplan–Meier survival curves were generated to visualize differences in survival according to biomarker cut-off values, and survival distributions were compared using the log-rank test. A two-sided *p*-value < 0.05 was considered statistically significant.

## 3. Results

In this study involving 600 ICU patients with sepsis, notable baseline differences were observed between survivors (*n* = 360) and non-survivors (*n* = 240). Non-survivors were significantly older (70.9 ± 13.2 vs. 62.4 ± 14.8 years, *p* < 0.001) and had higher rates of comorbidities, including diabetes mellitus (40.0% vs. 30.6%, *p* = 0.01), chronic kidney disease (25.0% vs. 13.3%, *p* < 0.001), COPD (15.8% vs. 9.7%, *p* = 0.03), ischemic heart disease (19.2% vs. 11.1%, *p* = 0.006), and malignancy (10.4% vs. 5.6%, *p* = 0.02). Septic shock was markedly more frequent among non-survivors (75.0% vs. 38.9%, *p* < 0.001). Disease severity scores were also higher in this group, with median APACHE II (24 vs. 17, *p* < 0.001) and SOFA scores (10 vs. 6, *p* < 0.001).

Metabolic and biochemical abnormalities were more pronounced among non-survivors, who exhibited significantly higher fasting plasma glucose (135 vs. 115 mg/dL, *p* < 0.001), triglyceride (145 vs. 130 mg/dL, *p* = 0.002), lactate (3.0 vs. 1.8 mmol/L, *p* < 0.001), CRP (180 vs. 160 mg/L, *p* = 0.004), and procalcitonin levels (5.8 vs. 3.5 ng/mL, *p* < 0.001), accompanied by lower HDL-C (33 vs. 38 mg/dL, *p* < 0.001) and albumin (2.7 ± 0.5 vs. 3.1 ± 0.5 g/dL, *p* < 0.001). Renal impairment was more prominent as reflected by higher creatinine (1.6 vs. 1.1 mg/dL, *p* < 0.001) (Table 1).

**Figure 2:**
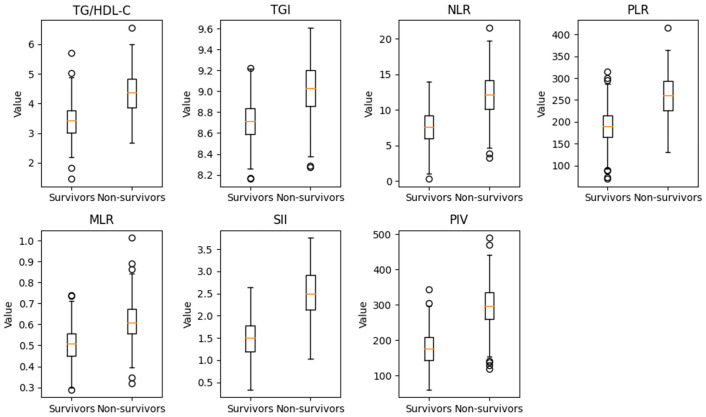
Distribution of triglyceride-to-HDL-C ratio (TG/HDL-C), triglyceride-glucose index (TGI), and inflammatory biomarkers (NLR, PLR, MLR, SII, PIV) in survivors and non-survivors with sepsis.

The distribution of metabolic and inflammatory biomarkers in survivors and non-survivors is shown in Table 2. Non-survivors exhibited markedly elevated values of both metabolic indices and immune-inflammatory markers. Specifically, the TG/HDL-C ratio (4.39 vs. 3.42, *p* < 0.001) and TGI (9.05 vs. 8.72, *p* < 0.001) were substantially higher in non-survivors, indicating more pronounced insulin resistance and metabolic stress. Inflammatory biomarkers showed a consistent upward trend in the non-survivor group: NLR (12.3 vs. 7.64, *p* < 0.001), PLR (260 vs. 190, *p* < 0.001), and MLR (0.62 vs. 0.50, *p* < 0.001) were all significantly elevated. Furthermore, composite inflammation indices demonstrated even stronger discriminatory patterns: SII (2.48 vs. 1.54, *p* < 0.001) and PIV (300 vs. 180, *p* < 0.001) were markedly higher in non-survivors (Table 2, Figure 2).

ROC analysis of metabolic and inflammatory biomarkers for predicting 28-day mortality is shown in Table 3. The TGI showed the highest discriminative power (AUC = 0.75, 95% CI: 0.71–0.79), followed by the TG/HDL-C ratio (AUC = 0.72, 95% CI: 0.68–0.76). Both indices demonstrated good sensitivity (73.8% and 70.4%, respectively) and specificity (70.0% and 68.1%, respectively).

**Figure 3:**
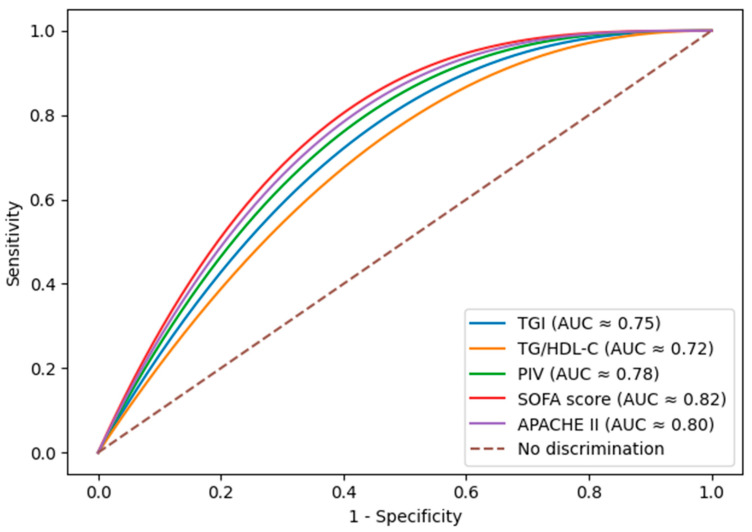
Receiver operating characteristic (ROC) curves of TGI, TG/HDL-C ratio, PIV, SOFA score, and APACHE II score for predicting 28-day mortality in ICU patients with sepsis.

Regarding inflammatory indices, the PIV achieved the best performance among cellular-based markers (AUC = 0.78, 95% CI: 0.74–0.82), outperforming the SII (AUC = 0.76) and NLR (AUC = 0.77). These values were comparable to the established severity scoring systems: APACHE II (AUC = 0.80) and SOFA (AUC = 0.82) (Table 3, Figure 3).

**Figure 4:**
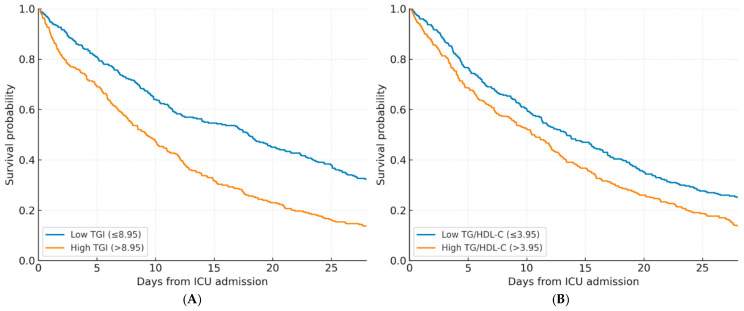
Kaplan–Meier survival curves stratified by (**A**) triglyceride–glucose index (TGI) using the threshold 8.95 and (**B**) triglyceride-to-HDL-C ratio (TG/HDL-C) using the threshold 3.95 for predicting 28-day survival among ICU patients with sepsis. Curves were compared with the log-rank test.

Figure 4 shows the Kaplan–Meier survival curves stratified according to the identified threshold values of the triglyceride–glucose index (TGI) (>8.95) and triglyceride-to–HDL-C ratio (TG/HDL-C) (>3.95) for predicting 28-day survival among ICU patients with sepsis. The survival analysis clearly demonstrates that patients with elevated TGI and TG/HDL-C values had significantly lower 28-day survival rates compared to those below these thresholds (*log-rank* *p* < 0.001 for both comparisons) (Figure 4).

In the univariate logistic regression analysis, several demographic, clinical, and laboratory variables were significantly associated with 28-day mortality. Older age, diabetes mellitus, chronic kidney disease, COPD, ischemic heart disease, malignancy, and the presence of septic shock were associated with an increased risk of mortality. In addition, higher disease severity scores (APACHE II and SOFA), elevated lactate and procalcitonin levels, and impaired renal function were also significantly associated with mortality. Among the investigated biomarkers, the metabolic indices TGI and TG/HDL-C ratio, as well as inflammatory markers NLR, PLR, MLR, SII, and PIV, were all significantly associated with 28-day mortality (Table 4).

Multivariable Cox regression analysis for predictors of 28-day mortality and incremental prognostic value is shown in Table 5. After adjustment for age, comorbidities, disease severity indicators, and laboratory parameters, TGI, TG/HDL-C ratio, and PIV remained independently associated with increased odds of 28-day mortality. Specifically, TGI > 8.95 was associated with a 44% increase in the odds of mortality (adjusted OR 1.44, 95% CI: 1.19–1.74, *p* < 0.001), while TG/HDL-C > 3.95 conferred a 31% increase (adjusted OR 1.31, 95% CI: 1.08–1.59, *p* = 0.005). PIV > 260 showed the strongest association (adjusted OR 1.49, 95% CI: 1.22–1.82, *p* < 0.001).

The combination of metabolic and inflammatory markers improved model discrimination beyond the SOFA score alone (AUC 0.86 vs. 0.82). Internal validation using bootstrap resampling demonstrated minimal optimism, with an optimism-corrected AUC of 0.85. The Hosmer–Lemeshow test indicated good calibration of the final model (*p* = 0.41) (Table 5).

**Figure 5:**
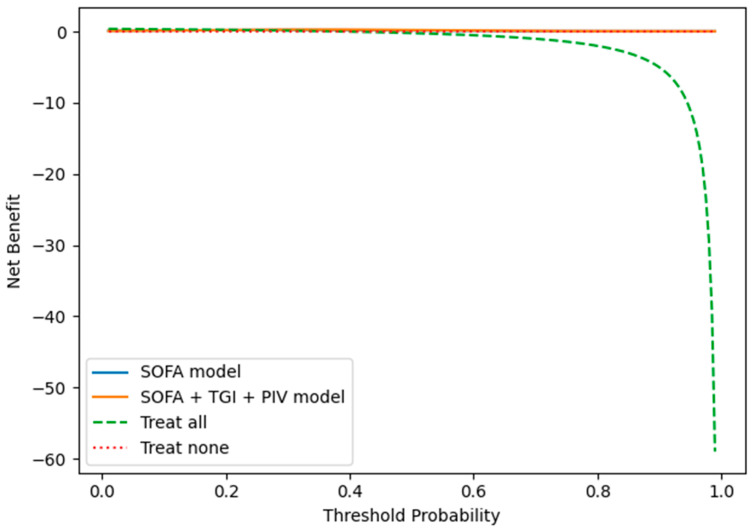
Decision curve analysis for predicting 28-day mortality.

Decision curve analysis further demonstrated that the combined SOFA + TGI + PIV model provided a higher net clinical benefit across a wide range of threshold probabilities compared with the SOFA model alone (Figure 5).

## 4. Discussion

In this large retrospective cohort of ICU patients with sepsis, we found that both the TGI and TG/HDL-C were significantly higher in non-survivors and independently associated with 28-day mortality. Among inflammatory indices, the PIV demonstrated strong prognostic performance, comparable to the SOFA and APACHE II scores. Furthermore, integrating TGI and PIV into conventional scoring systems modestly improved mortality prediction accuracy, suggesting that metabolic and inflammatory pathways jointly contribute to sepsis pathophysiology and outcomes.

Our findings align with several recent studies demonstrating that elevated TGI levels are strongly linked to poor outcomes in sepsis. Zheng et al. first showed that a higher TGI was independently associated with increased in-hospital mortality among septic patients in the MIMIC-IV database [6]. Fang et al. and Cao et al. reported that elevated TGI values predicted sepsis-associated acute kidney injury (SA-AKI), prolonged ICU stay, and increased 30-day mortality [7,8]. Lou et al. also observed that higher TGI levels correlated with organ dysfunction and mortality risk in critically ill patients with sepsis, emphasizing its value as a simple marker of insulin resistance and metabolic stress [9].

Consistent with these studies, our analysis identified a TGI cut-off of 8.95, above which the risk of 28-day mortality increased by approximately 42% after adjustment for confounders. This finding supports the hypothesis that insulin resistance-related metabolic dysregulation plays a key role in sepsis progression and outcome. Additionally, Lv et al. showed that this relationship persisted regardless of obesity status, suggesting that metabolic stress during infection, rather than baseline adiposity, drives adverse prognosis [10]. The longitudinal analysis by Ning et al. further corroborates our results, showing that dynamic changes in TGI trajectories are associated with sepsis subphenotypes characterized by higher mortality [11]. Because metabolic indices such as TGI may be influenced by underlying metabolic disorders, including diabetes mellitus, we additionally adjusted our models for major comorbidities. Even after controlling for these potential confounders, TGI remained an independent predictor of mortality.

We also found that the TG/HDL-C ratio was an independent predictor of mortality in sepsis. This association is biologically plausible given the dual role of lipids in immune defense and inflammation. Hofmaenner et al. highlighted the critical function of lipoproteins in binding and neutralizing bacterial toxins; therefore, reduced HDL-C levels may impair endotoxin clearance and exacerbate systemic inflammation [4]. In our cohort, non-survivors had significantly lower HDL-C and higher triglyceride levels, consistent with the dyslipidemic profile reported in critically ill patients.

Our findings are in line with Che et al., who demonstrated that TG/HDL-C ratio reflects atherogenic dyslipidemia and predicts cardiovascular events, and Peng et al., who showed that elevated TG/HDL-C ratios were associated with increased mortality in COVID-19 patients [12,14]. Wang et al. also recently confirmed that both TG/HDL-C and TGI are linked to disease severity in acute pancreatitis, supporting the broader prognostic relevance of these indices in critical illness [16]. Taken together, these results suggest that TG/HDL-C ratio is a valuable, easily obtainable biomarker that integrates metabolic and inflammatory risk in sepsis.

Despite the recognized role of lipid metabolism in sepsis pathophysiology, clinical trials targeting lipid pathways have produced inconsistent results. Several studies have investigated the potential benefit of lipid-lowering agents, particularly statins, due to their pleiotropic anti-inflammatory and immunomodulatory effects. Experimental and observational studies suggested that statins may attenuate endothelial dysfunction, reduce cytokine release, and improve microvascular perfusion during sepsis. However, randomized clinical trials have largely failed to demonstrate a consistent survival benefit. For example, the SAILS trial and the HARP-2 trial evaluating statin therapy in sepsis-associated acute respiratory distress syndrome did not show significant reductions in mortality or organ failure. These findings highlight the complex relationship between lipid metabolism and immune responses in sepsis. While metabolic dysregulation clearly contributes to disease severity, pharmacological modulation of lipid pathways has not yet translated into effective therapeutic strategies. In this context, metabolic biomarkers such as the triglyceride–glucose index may serve primarily as prognostic indicators rather than direct therapeutic targets.

Beyond metabolic dysregulation, inflammation remains central to sepsis pathophysiology. Consistent with previous reports, our study found that NLR, SII, and PIV were significantly elevated in non-survivors. Hwang et al. and Shi et al. reported that higher NLR values were independently associated with in-hospital mortality among septic patients [21,22]. Zhang et al. demonstrated that elevated SII predicted 28-day mortality in sepsis-associated AKI [28], corroborating our observation that SII levels were markedly higher in non-survivors.

Among inflammatory indices, PIV showed the highest predictive performance (AUC = 0.78) for 28-day mortality, comparable to the SOFA score. This result echoes findings from Bilgin et al., who demonstrated the prognostic power of PIV in acute coronary syndrome, and from Zhou et al., who confirmed its association with mortality in sepsis-related AKI [30,32]. Furthermore, Giri et al. and Zhao et al. demonstrated the predictive value of PIV in COPD and pulmonary embolism, respectively, underscoring its potential as a universal inflammatory marker across critical illnesses [31,33]. These results collectively indicate that PIV effectively captures the interplay between innate immune activation and adaptive suppression that characterizes severe sepsis.

One of the novel aspects of our study is the combined evaluation of metabolic (TGI, TG/HDL-C) and inflammatory (PIV, SII, NLR) markers. While previous studies have investigated these parameters separately, our results demonstrate that adding TGI and PIV to the SOFA score modestly enhanced prognostic accuracy. This suggests that metabolic and immune dysfunctions act synergistically to worsen outcomes in sepsis. Given the interdependence of hyperglycemia, lipotoxicity, and immune dysregulation, these markers may reflect distinct yet complementary pathophysiological axes contributing to multiorgan failure.

Our findings have potential clinical implications. Since TGI and TG/HDL-C can be readily calculated from routine laboratory data, they could serve as cost-effective, non-invasive tools for early risk stratification in ICU settings. Identifying high-risk patients based on these indices could facilitate timely interventions, personalized glycemic–lipid management, and optimization of nutritional and metabolic support. Moreover, combining PIV with TGI may provide a more holistic assessment of metabolic–inflammatory status than traditional scoring systems alone.

The main strengths of this study include its large sample size, detailed biochemical data, and comprehensive evaluation of both metabolic and inflammatory markers. To strengthen the robustness of the predictive model, internal validation using bootstrap resampling and calibration analysis using the Hosmer–Lemeshow test were performed. However, certain limitations should be acknowledged. The retrospective single-center design limits causal inference, and residual confounding cannot be excluded despite multivariate adjustments. To minimize potential bias, we applied a stepwise analytical strategy including univariate screening followed by multivariate logistic regression modeling with adjustment for clinically relevant confounders. To reduce potential over-optimism of predictive performance estimates, internal validation using bootstrap resampling was applied. Additionally, we lacked serial measurements of biomarkers, which could provide insight into their dynamic changes during sepsis progression. Finally, external validation in multicenter cohorts is warranted to confirm generalizability.

## 5. Conclusions

In conclusion, both the TGI and TG/HDL-C ratio are independent predictors of short-term mortality in ICU patients with sepsis. The PIV demonstrated comparable prognostic accuracy to established clinical scores. Combining these metabolic and inflammatory indices with the SOFA score enhanced risk prediction, underscoring the integrated role of metabolic and immune dysfunctions in sepsis pathophysiology. Prospective multicenter studies are needed to validate these findings and explore their potential integration into clinical decision-making tools.

## Figures and Tables

**Table 1:** Baseline characteristics of survivors and non-survivors (*n* = 600).

Variables	Survivors (*n* = 360)	Non-Survivors (*n* = 240)	*p*-Value
Age, years, mean ± SD	62.4 ± 14.8	70.9 ± 13.2	<0.001
Male sex, *n* (%)	210 (58.3)	150 (62.5)	0.32
BMI, kg/m^2^, mean ± SD	26.1 ± 4.3	25.4 ± 4.8	0.08
Diabetes mellitus, *n* (%)	110 (30.6)	96 (40.0)	0.01
Hypertension, *n* (%)	165 (45.8)	122 (50.8)	0.24
Chronic kidney disease, *n* (%)	48 (13.3)	60 (25.0)	<0.001
COPD, *n* (%)	35 (9.7)	38 (15.8)	0.03
Ischemic heart disease, *n* (%)	40 (11.1)	46 (19.2)	0.006
Chronic liver disease, *n* (%)	18 (5.0)	22 (9.2)	0.04
Malignancy, *n* (%)	20 (5.6)	25 (10.4)	0.02
Primary infection site—Respiratory, *n* (%)	170 (47.2)	136 (56.7)	0.02
Primary infection site—Abdominal, *n* (%)	110 (30.6)	66 (27.5)	0.44
Primary infection site—Other, *n* (%)	80 (22.2)	38 (15.8)	0.06
Septic shock, *n* (%)	140 (38.9)	180 (75.0)	<0.001
APACHE II score, median (IQR)	17 (14–21)	24 (20–29)	<0.001
SOFA score, median (IQR)	6 (4–8)	10 (8–13)	<0.001
Fasting plasma glucose, mg/dL, median (IQR)	115 (98–135)	135 (112–160)	<0.001
Triglycerides, mg/dL, median (IQR)	130 (102–160)	145 (118–180)	0.002
HDL-C, mg/dL, median (IQR)	38 (32–45)	33 (28–40)	<0.001
Total cholesterol, mg/dL, median (IQR)	158 (140–180)	150 (132–170)	0.01
LDL-C, mg/dL, median (IQR)	90 (76–105)	84 (70–100)	0.02
Lactate, mmol/L, median (IQR)	1.8 (1.3–2.4)	3.0 (2.3–4.2)	<0.001
CRP, mg/L, median (IQR)	160 (110–210)	180 (135–240)	0.004
Procalcitonin, ng/mL, median (IQR)	3.5 (1.5–7.0)	5.8 (3.0–12.0)	<0.001
Albumin, g/dL, mean ± SD	3.1 ± 0.5	2.7 ± 0.5	<0.001
Creatinine, mg/dL, median (IQR)	1.1 (0.9–1.5)	1.6 (1.2–2.3)	<0.001
Neutrophils, ×10^9^/L, median (IQR)	8.4 (6.8–10.2)	9.6 (7.8–11.4)	<0.001

**Table 2:** Distribution of metabolic and inflammatory biomarkers in survivors and non-survivors (*n* = 600).

Variables	Survivors (*n* = 360)	Non-Survivors (*n* = 240)	*p*-Value
TG/HDL-C ratio	3.42 (2.80–4.10)	4.39 (3.60–5.10)	<0.001
Triglyceride–glucose index (TyG)	8.72 (8.40–9.05)	9.05 (8.75–9.42)	<0.001
Neutrophil-to-lymphocyte ratio (NLR)	7.64 (5.50–10.20)	12.30 (9.40–15.80)	<0.001
Platelet-to-lymphocyte ratio (PLR)	190 (150–240)	260 (200–320)	<0.001
Monocyte-to-lymphocyte ratio (MLR)	0.50 (0.38–0.63)	0.62 (0.48–0.80)	<0.001
Systemic immune-inflammation index (SII)	1.540 (1.200–2.020)	2.480 (1.950–3.210)	<0.001
Platelet inflammatory value (PIV)	180 (140–240)	300 (240–380)	<0.001

**Table 3:** ROC analysis of metabolic and inflammatory biomarkers for predicting 28-day mortality.

Biomarker	AUC (95% CI)	Cut-Off	Sensitivity (%)	Specificity (%)	PPV (%)	NPV (%)
TG/HDL-C ratio	0.72 (0.68–0.76)	3.95	70.4	68.1	61.2	76.0
TGI	0.75 (0.71–0.79)	8.95	73.8	70.0	63.9	78.6
NLR	0.77 (0.73–0.81)	10.5	72.1	74.2	66.7	78.9
PLR	0.70 (0.66–0.75)	235	65.8	70.8	60.2	75.4
MLR	0.69 (0.64–0.73)	0.58	63.3	68.3	58.1	72.9
SII	0.76 (0.72–0.80)	2150	71.2	73.3	65.5	77.8
PIV	0.78 (0.74–0.82)	260	74.6	73.3	67.9	79.2
APACHE II	0.80 (0.76–0.83)	21	76.7	74.2	69.3	80.7
SOFA	0.82 (0.78–0.85)	9	78.8	76.9	72.1	82.5

**Table 4:** Univariate logistic regression analysis of demographic, clinical, metabolic, and inflammatory variables associated with 28-day mortality in ICU patients with sepsis.

Variable	OR	95% CI	*p*-Value
Age (per 10-year increase)	1.38	1.24–1.53	<0.001
Male sex	1.14	0.92–1.42	0.23
BMI (per kg/m^2^)	0.97	0.94–1.01	0.12
Diabetes mellitus	1.36	1.07–1.73	0.012
Hypertension	1.18	0.94–1.47	0.15
Chronic kidney disease	1.79	1.36–2.36	<0.001
COPD	1.41	1.03–1.92	0.031
Ischemic heart disease	1.49	1.10–2.02	0.011
Chronic liver disease	1.45	1.03–2.05	0.034
Malignancy	1.57	1.10–2.25	0.014
Septic shock	2.81	2.17–3.64	<0.001
APACHE II score (per point)	1.10	1.07–1.12	<0.001
SOFA score (per point)	1.23	1.18–1.27	<0.001
Lactate (per mmol/L)	1.31	1.22–1.41	<0.001
Procalcitonin (log-transformed)	1.22	1.12–1.33	<0.001
CRP (per 10 mg/L increase)	1.04	1.01–1.06	0.004
Albumin (per g/dL)	0.69	0.59–0.80	<0.001
Creatinine (per mg/dL)	1.28	1.16–1.41	<0.001
Triglyceride–glucose index (TGI)	1.61	1.36–1.91	<0.001
TG/HDL-C ratio	1.42	1.24–1.63	<0.001
NLR	1.05	1.03–1.07	<0.001
PLR	1.02	1.01–1.03	<0.001
MLR	1.52	1.21–1.90	<0.001
SII	1.15	1.08–1.22	<0.001
PIV	1.20	1.13–1.28	<0.001

**Table 5:** Multivariable logistic regression analysis for predictors of 28-day mortality and model performance.

Variable	Adjusted OR	95% CI	*p*-Value
Age (per 10-year increase)	1.32	1.18–1.47	<0.001
Male sex	1.09	0.86–1.37	0.47
Diabetes mellitus	1.21	0.95–1.54	0.12
Chronic kidney disease	1.39	1.05–1.84	0.021
Septic shock	2.08	1.57–2.75	<0.001
SOFA score (per point)	1.19	1.13–1.24	<0.001
Lactate (per 1 mmol/L)	1.22	1.13–1.32	<0.001
Albumin (per g/dL)	0.74	0.63–0.88	<0.001
Creatinine (per mg/dL)	1.17	1.05–1.31	0.006
Procalcitonin (per 1 ng/mL, log-transformed)	1.16	1.07–1.26	<0.001
TGI (>8.95)	1.44	1.19–1.74	<0.001
TG/HDL-C ratio (>3.95)	1.31	1.08–1.59	0.005
PIV (>260)	1.49	1.22–1.82	<0.001
**Model performance and internal validation**			
**Model**	**AUC (95% CI)**	**Bootstrap-corrected AUC**	**ΔAUC vs.** **SOFA alone**
SOFA alone	0.82 (0.78–0.85)	0.81	—
SOFA + TGI	0.84 (0.80–0.87)	0.83	+0.02
SOFA + TG/HDL-C	0.83 (0.79–0.86)	0.82	+0.01
SOFA + PIV	0.85 (0.81–0.88)	0.84	+0.03
SOFA + TGI + PIV	0.86 (0.82–0.89)	0.85	+0.04
**Model validation**			
**Metric**	**Value**		
Apparent AUC	0.86		
Bootstrap-corrected AUC	0.85		
Hosmer–Lemeshow χ^2^	7.42		
Hosmer–Lemeshow *p*	0.41		

## Data Availability

The data supporting the findings of this study are available from the corresponding author upon reasonable request. The data are not publicly available due to their use in ongoing analyses and model development.
